# The Association between Contrast Dose and Renal Complications Post PCI across the Continuum of Procedural Estimated Risk

**DOI:** 10.1371/journal.pone.0090233

**Published:** 2014-03-13

**Authors:** Judith Kooiman, Milan Seth, David Share, Simon Dixon, Hitinder S. Gurm

**Affiliations:** 1 Department of Thrombosis and Hemostasis and Department of Nephrology, Leiden University Medical Center, Leiden, Zuid-Holland, The Netherlands; 2 Department of Internal Medicine, Division of Cardiovascular Medicine, University of Michigan, Ann Arbor, Michigan, United States of America; 3 Blue Cross Blue Shield of Michigan, Detroit, Michigan, United States of America; 4 Beaumont Hospital, Royal Oak, Michigan, United States of America; Ferrarotto Hospital, University of Catania, Italy

## Abstract

**Background:**

Prior studies have proposed to restrict the contrast volume (CV) to <3x calculated creatinine clearance (CCC), to prevent contrast induced nephropathy (CIN) post percutaneous coronary interventions (PCI). The predictive value of this algorithm for CIN and therefore the benefit of this approach in high risk patients has been questioned. The aim of our study was to assess the association between contrast dose and the occurrence of CIN in patients at varying predicted risks of CIN and baseline CCC following contemporary PCI.

**Methods:**

Consecutive patients undergoing PCI between 2010–2012 were included. Baseline risk of CIN was calculated using a previously validated risk tool. High contrast dose was defined as CV/CCC >3. Likelihood ratio tests were used to evaluate whether the effect of a high contrast dose on the risk of CIN and nephropathy requiring dialysis (NRD) varied across the spectrum of baseline predicted risk.

**Results:**

Of the 82,120 PCI included in our analysis, 25% were performed using a high contrast dose. Patients treated with a high compared with a low contrast dose were at increased risks of CIN and NRD, throughout the entire range of baseline predicted risk and CCC in our population. The effect size of a high contrast dose on risks of both outcomes varied significantly with baseline predicted CIN risk and CCC (CIN p = 0.004, NRD p<0.001 for adding interactions), and was largest for patients with predicted CIN risk <10% and pre-existing chronic kidney disease.

**Conclusions:**

The use of a high contrast dose is associated with increased risks of CIN and NRD across the continuum of baseline predicted risk and CCC. Efforts to reduce contrast dose may therefore be effective in preventing renal complications in all patients undergoing PCI.

## Introduction

Contrast media induced renal complications are common among patients undergoing percutaneous coronary interventions (PCI) [1]. Contrast induced nephropathy (CIN) and the need for dialysis (NRD) post PCI have been associated with increased early and long-term mortality rates, and add significantly to healthcare expenses [1], [2]. Current guidelines support multiple strategies for prophylaxis of CIN including adequate hydration, minimization of contrast dose and the use of iso-osmolar or certain low-osmolar contrast media[3]–[7].

Use of renal function based contrast dosing with the total contrast volume (CV) restricted to less than thrice the calculated creatinine clearance (CCC) has been suggested as a practical strategy to reduce the risk of CIN [3]. However, the predictive value of this dosing algorithm for CIN and hence the benefit of this approach in patients at high risk of renal complications post PCI has been debated [8].

We recently reported an accurate prediction model for the risk of renal complications in patients undergoing PCI [9]. This model estimates the risk of CIN and NRD based on pre-procedural variables, has a higher discriminative power than other commonly used prediction models, and can therefore be used to study the effect of a high contrast dose on the occurrence of CIN in patients with varying baseline risks of renal complications.

The aim of our current study was to assess the impact of high contrast dose (CV/CCC >3) on the risk of renal complications across the continuum of pre-procedural predicted risk of CIN in a large cohort of patients undergoing contemporary PCI.

## Methods

Our study population comprised consecutive patients undergoing PCI between January 2010 and September 2012 across 47 hospitals in Michigan participating in the Blue Cross Blue Shield of Michigan Cardiovascular Consortium (BMC2). Hence, this cohort consists of patients other than the population in which the effect of renal function-based contrast dosing on the risk of renal complications was originally assessed (who underwent PCI between 2007 and 2008) [3]. BMC2 is a quality improvement collaborative that tracks the inpatient outcome of consecutive patients undergoing PCI at all non-federal hospitals in the State of Michigan. The details of the BMC2 and its data collection and auditing process have been described previously [10], [11]. Procedural data on all consecutive patients undergoing PCI at participating hospitals are collected using standardized data collection forms. Collected data include clinical and demographic patient characteristics, procedural, and angiographic characteristics, as well as medications used before, during, and after PCI, and in-hospital outcomes. All data elements have been prospectively defined, and the protocol is approved by local institutional review boards at each of the participating hospitals. In addition to a random audit in 2% of all PCI procedures, medical records of all patients undergoing multiple procedures or coronary artery bypass grafting and of patients who died in the hospital are reviewed routinely to ensure data accuracy.

Patients who were already on dialysis at the time of PCI, those with missing serum creatinine values pre or post procedurally, and those who died in the catheterization laboratory were excluded from outcome analysis. Patients with missing values for weight, gender, or CV were also excluded as these variables were needed to determine whether a patient received a high contrast dose. The type and volume of contrast media and hydration protocols used were as per the operator preference guided by institutional policy and practice.

### Study Endpoints

CIN was defined as an acute decline in renal function post PCI resulting in an absolute increase in serum creatinine ≥0.5 mg/dL from baseline [12]. Baseline creatinine values were collected within a month prior to PCI. Among patients who had multiple assessments of serum creatinine in the month prior to PCI, the value closest to the time of the procedure was considered as the baseline value. Peak creatinine was defined as the highest value of creatinine in the week following the procedure or during the hospitalization following PCI and was ascertained as per local clinical practice. Time between PCI and peak creatinine was at least 1 day, and varied depending on length of hospital stay. The secondary endpoint for the study was NRD defined as a new, unplanned need for dialysis during hospitalization due to progression of chronic kidney disease post PCI.

High contrast dose was defined as administration of a CV thrice the CCC. CCC was calculated using the Cockgroft-Gault equation [13].

### Statistical Analyses

Continuous variables are presented as mean with standard deviation and categorical variables as percentages, with standardized differences between groups presented for both types of variables as percentages. Unless otherwise stated, student t-tests for continuous and Chi-squared tests for categorical variables were utilized for univariate comparisons.

Baseline estimated risk of CIN was calculated using the BMC2 CIN prediction tool [9]. Multivariate logistic regression models with CIN and NRD as outcomes were developed including high contrast dose, baseline estimated CIN risk, CCC, and other baseline clinical covariates as main effect terms. To investigate potential effect modification of baseline predicted CIN risk on CIN and NRD rates associated with a high contrast dose, regression models adding two and three way interaction terms involving baseline estimated CIN risk (logit transformed linear and quadratic terms) and CCC with high contrast dose were fitted to the data. A stepwise selection algorithm optimizing the Akaike Information Criteria was used to select an optimal model. The selected models including interactions were then compared to the base model using likelihood ratio tests to assess whether inclusion of interactions significantly improved the fit of the model.

Model predicted relative risks for CIN and NRD comparing high with low contrast dosages were plotted over a range of CCC and baseline predicted risk of CIN, to demonstrate the extent and implications of effect modification.

All analyses were performed in R version 2.14.1 using freely distributed contributed packages [14], [15].

## Results

Our study cohort comprised 82,120 (85%) of the 96,753 PCI procedures performed across Michigan between January 2010 through December 2012. Of the 14,633(15%) procedures that were excluded from the analysis, 2,251 (15%) patients were already on dialysis at the time of the procedure, 11,997 (82%) had missing serum creatinine values prior to (n = 2,229 (15%)) or following PCI (n = 9,907 (68%)), with 139 patients missing both pre and post procedural creatinine values. Additionally, 466 patients lacked information on CV, 99 on bodyweight, and 1 patient on gender. As all of these variables were needed to determine whether a patient received a high contrast dose, patients missing one or more of these data elements were excluded.

Table 1 reports characteristics at baseline of study patients categorized to the use of either a low or a high contrast dose at time of PCI (CV/CCC < = 3 and CV/CCC >3). In this cohort, 20,915(25.3%) patients received a high contrast dose who had a greater burden of comorbidities, a higher baseline estimated CIN risk, and were more likely to have preexisting chronic kidney disease.

**Table 1:** Baseline characteristics of patients treated with high versus low contrast dose.

Characteristic	CV/CCC ≤3	CV/CCC >3	P-value	Standardized difference (%)
N (procedures)	61.205 (74.5%)	20.915 (25.5%)	NA	NA
BMI	31.48±7.83	27.89±5.63	<0.001	52.54
Age	62.16±11.36	73.26±10.53	<0.001	101.28
Creatinine clearance (CCC)	106.24±42.83	59.10±22.87	<0.001	137.31
Contrast volume (ml)	171.84±63.15	248.90±88.46	<0.001	100.28
Predicted CIN risk (%)	2.10±4.99	5.23±8.91	<0.001	43.32
Female gender	18.838/61.205 (30.8%)	9.039/20.915 (43.2%)	<0.001	25.98
Race - White	53.502/61.205 (87.4%)	17.725/20.915 (84.7%)	<0.001	7.71
Race - Black or African American	6.189/61.205 (10.1%)	2.655/20.915 (12.7%)	<0.001	8.13
Current/recent smoker (w/in 1 year)	20.138/61.178 (32.9%)	4.099/20.906 (19.6%)	<0.001	30.60
Hypertension	51.108/61.185 (83.5%)	18.726/20.905 (89.6%)	<0.001	17.79
Prior MI	20.910/61.196 (34.2%)	7.645/20.910 (36.6%)	<0.001	5.01
Prior heart failure	7.924/61.185 (13.0%)	4.696/20.907 (22.5%)	<0.001	25.11
Prior PCI	27.440/61.200 (44.8%)	9.087/20.913 (43.5%)	<0.001	2.79
Prior CABG	9.642/61.187 (15.8%)	5.668/20.912 (27.1%)	<0.001	27.92
Cerebrovascular disease	7.821/61.187 (12.8%)	4.652/20.907 (22.3%)	<0.001	25.11
Peripheral arterial disease	8.447/61.190 (13.8%)	4.937/20.909 (23.6%)	<0.001	25.35
Diabetes mellitus	22.697/61.198 (37.1%)	7.715/20.912 (36.9%)	0.614	0.40
**CAD presentation/Evaluation**				
No symptom. no angina	4.172/61.185 (6.8%)	1.329/20.906 (6.4%)	0.021	1.86
Symptom unlikely ischemic	1.352/61.185 (2.2%)	494/20.906 (2.4%)	0.197	1.03
Stable angina	9.701/61.185 (15.9%)	3.215/20.906 (15.4%)	0.102	1.31
Unstable angina	24.129/61.185 (39.4%)	7.957/20.906 (38.1%)	<0.001	2.82
Non-STEMI	12.223/61.185 (20.0%)	4.619/20.906 (22.1%)	<0.001	5.20
STEMI or equivalent	9.608/61.185 (15.7%)	3.292/20.906 (15.7%)	0.881	0.12
Cardiogenic shock w/in 24 hours	759/61.187 (1.2%)	592/20.909 (2.8%)	<0.001	11.28
Cardiac arrest w/in 24 hours	1.037/61.166 (1.7%)	460/20.901 (2.2%)	<0.001	3.66
**PCI Indication**				
Immediate PCI for STEMI	8.451/61.195 (13.8%)	2.912/20.909 (13.9%)	0.672	0.34
PCI for STEMI (unstable. >12 hrsfrom Sx onset)	421/61.195 (0.7%)	223/20.909 (1.1%)	<0.001	4.06
PCI for STEMI (Stable. >12 hrsfrom Sx onset)	218/61.195 (0.4%)	85/20.909 (0.4%)	0.301	0.82
Staged PCI	4.222/61.195 (6.9%)	870/20.909 (4.2%)	<0.001	12.00

Data are presented as mean (SD), or N (%) unless stated otherwise.

Abbreviations: BMI = body mass index, CIN = contrast induced nephropathy, CV/CCC = contrast volume/calculated creatinine clearance, MI = myocardial infarction, PCI = percutaneous coronary intervention, CABG = coronary artery bypass graft, CAD = coronary artery disease, STEMI = ST-elevation myocardial infarction.

**Figure 1:**
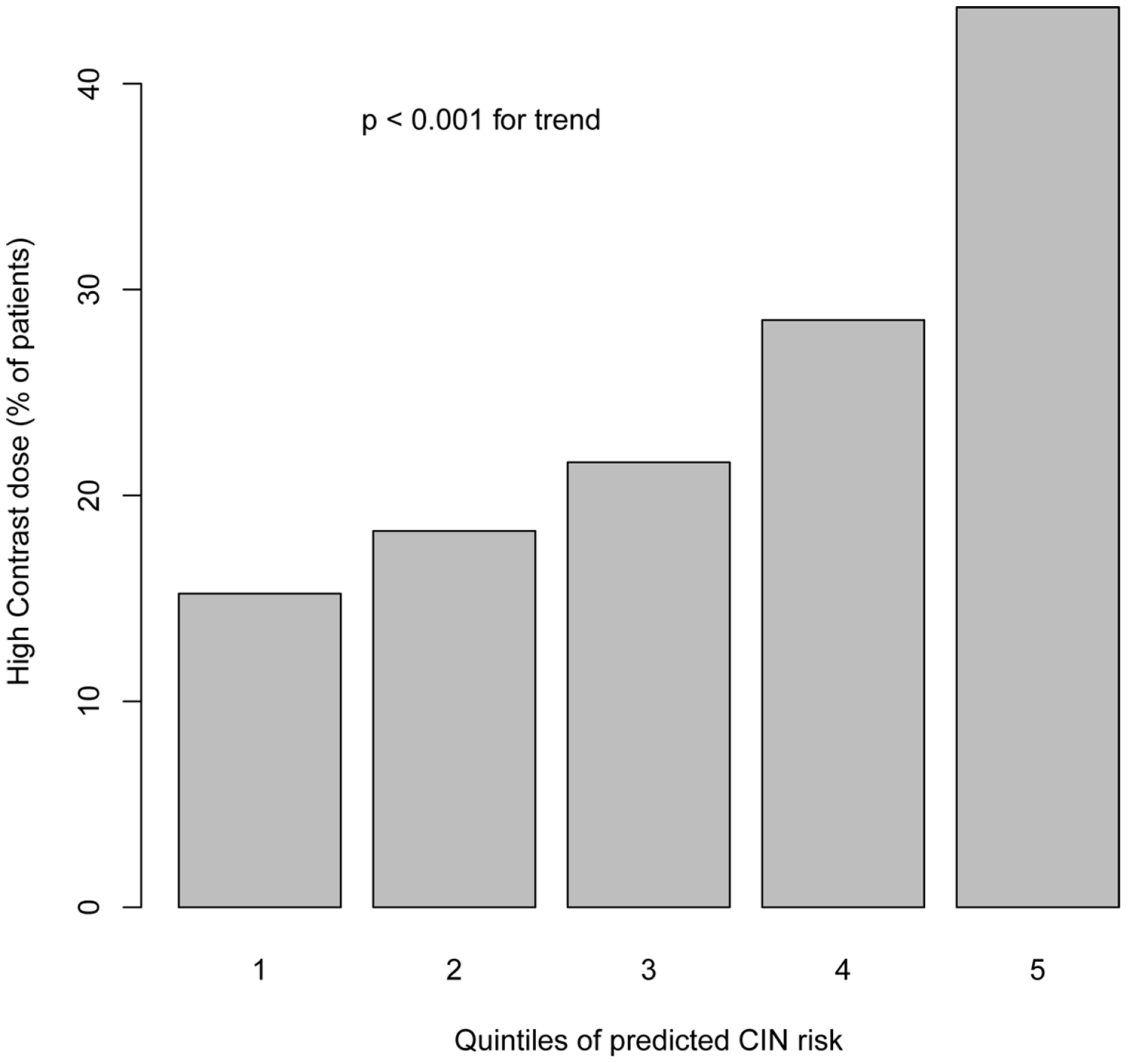
Proportions of patients treated with high dose contrast (Contrast volume/calculated creatinine clearance >3) across the quintiles of predicted risk of contrast induced nephropathy.

The median predicted risk of CIN of the cohort was 0.54% (range = 0–80.4%, IQR = 0.08%–2.43%), and 90% of patients had a predicted risk of less than 7.92%. Patients at higher predicted risk of CIN were more likely to be treated with a high contrast dose (p<0.001, Figure 1).

CIN occurred in 2,146/82,120 (2.61%, 95% CI 2.51–2.72%) patients, and NRD in 308/82,120 (0.37%, 95% CI 0.33–0.42%). The median baseline CIN risk estimate, calculated using the risk tool, was 11.6% (IQR 3.1–25.5%) among patients developing CIN, and 24.9% (10.9–36.6%) among those with NRD. Of patients with CIN, 1,144/2,146 (53.3%, 95% CI 51.2–55.4%) received a high contrast doses, as did 211/308 (68.5%, 95% CI 63.0–73.7%) patients with NRD.

In regression models adjusting for baseline predicted CIN risk and CCC, high contrast dose was significantly associated with increased rates of both CIN (OR = 1.61, 95% CI 1.46–1.79, P<.001) and NRD (OR = 1.65, 95% CI 1.24–2.21, P<.001), indicating that high contrast dose is an independent predictor of these outcomes. Within a multivariate model adjusting for baseline clinical covariates (age, gender, recent heart failure, cardiogenic shock, cardiac arrest, CAD presentation, PCI status and indications), the effect of high contrast dose on the risk of CIN and NRD was consistent for both outcomes (CIN OR = 1.77, 95% CI 1.58–1.98, P<.001, NRD OR = 1.92, 95% CI 1.13–2.16, P<0.01).

Regression models were developed to investigate potential effect modification of baseline predicted CIN risk and baseline CCC on the association between high contrast dose and the risks of CIN and NRD. The fit of the models were significantly improved compared with the base models after adding of interaction terms involving baseline estimated CIN risk and CCC (Likelihood ratio test: LR 28 on 11 df, p = 0.004 for CIN,LR = 29 on 6 df, p<0.001 for NRD). This indicates that the effect of high contrast dose on the risks of both CIN and NRD varied significantly across the spectrum of predicted CIN risk and baseline CCC.

**Figure 2:**
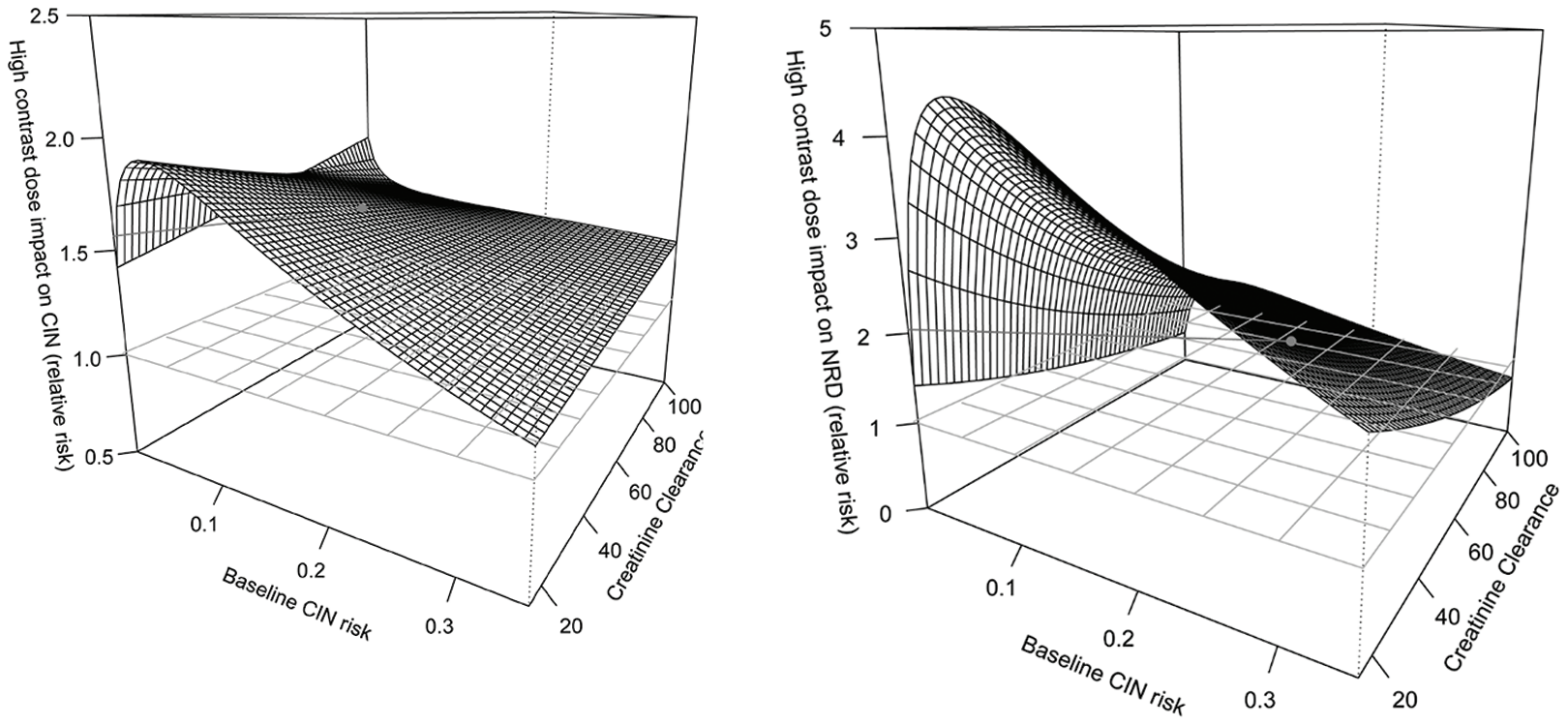
**A.** The relative risk of contrast induced nephropathy in association with high contrast dose across the continuum of predicted risk of contrast induced nephropathy among patients undergoing PCI. **B.** The relative risk of nephropathy requiring dialysis in association with high contrast dose across the continuum of predicted risk of contrast induced nephropathy among patients undergoing PCI.

Figure 2 depicts the model predicted relative risk of CIN (Figure 2A) and NRD (Figure 2B) for high versus a low contrast dose across the spectrum of baseline predicted risk and CCC. Both figures demonstrate an increased risk of renal complications post PCI associated with a high contrast dose regardless of a patient’s baseline predicted risk or CCC, although the effect of a high contrast dose was most pronounced in those at lower predicted risk. The points on the graphed surfaces highlighted in red represent the estimated relative risks of high versus a low contrast dose at the median baseline risk and creatinine clearance values for patients with CIN (risk: 11.6%, CCC: 57 ml/min) and NRD (risk: 24.9%, CCC: 42.6 ml/min). The relative risk associated with high a contrast dose at this point is 1.56 (95% CI 1.37–1.76) for CIN, and 2.05 (95% CI 1.35–2.75) for NRD.

## Discussion

The key finding of our study is that the use of a high contrast dose is associated with increased risks of CIN and NRD across the continuum of predicted risk and CCC. Efforts to reduce the contrast dose may therefore be effective in preventing renal complications in all patients undergoing PCI. Especially in patients with pre-existing renal failure, restricting the contrast dose might result in lower rates of NRD.

Our findings significantly add to extend prior observations in this field. Work from many groups including ours has highlighted the role of high contrast dose as a risk factor for CIN and NRD in patients undergoing invasive cardiac procedures [3], [16], [17]. We have now demonstrated that regardless of baseline CCC and predicted risk of renal complications, the use of a high contrast dose is associated with increased risks of CIN and NRD post PCI. These results are in line with the results of two previous smaller studies concluding a high contrast dose to be associated with an increased risk of CIN throughout the continuum of baseline renal function [18], [19]. These studies however, did not analyze the effect of baseline predicted risk on the association between a high contrast dose and renal complications post PCI. Our results demonstrated the effect of a high contrast dose on the risk of CIN and NRD to be most pronounced in those at lower predicted risk.

Efforts to improve outcomes of patients undergoing PCI for acute myocardial infarction or cardiogenic shock have traditionally focused on enhancing myocardial perfusion and hemodynamic support [20], [21]. Restriction of contrast dose might be another important strategy to improve patient outcome after PCI, as our study findings suggest that the use of high contrast dosages is associated with an increased risk of CIN in all patients undergoing PCI. Limiting the CV to less than thrice the CCC may be even more important in patients at high risk of CIN, in terms of an absolute risk reduction, but also in those with pre-existing chronic kidney disease (i.e. eGFR <60 ml/min) in whom the effect of a high contrast dose on the risks of CIN was more profound compared to those without renal impairment. Our study findings also suggest that efforts to limit contrast dose in patients undergoing PCI may be helpful in reducing the risk of NRD, a complication which although rare, has not declined in the last few years among patients undergoing PCI, regardless of the introduction of less nephrotoxic contrast agents.

Patients at high predicted risk of CIN were more likely to receive a high contrast dose in our study. These patients might more frequently have comorbidity than patients receiving a low contrast dose, like peripheral artery disease, or an altered coronary vasculature due to prior PCI or prior coronary artery bypass graft resulting in more complex PCI procedures, requiring higher contrast volumes. Additionally, as a high contrast dose (CV/CCC >3) is also driven by impaired renal function, the frequent use of a high contrast dose in patients at high risk might also be explained by comorbidity associated with chronic kidney disease, increasing the baseline predicted risk of CIN. Measures to reduce contrast dose are well recognized in literature but most of the work on contrast preservation has been performed in elective or stable patients, not those with acute myocardial infarction. Therefore, further research of the preventive effect of contrast preservation on the risk of renal complications in patients undergoing emergent PCI is warranted [22].

Our study findings must, however, be interpreted with certain qualifications. Our study associations, while strong do not support causality. However, it is unlikely that a randomized trial would ever be performed to evaluate the impact of high versus low contrast dose on the risk of renal complications. Since the findings appear biologically plausible and statistically robust, measures to limit contrast dose especially in high risk patients should be considered to reduce the risk of CIN and NRD. We have used the term CIN, although the role of contrast media in all patients who develop acute kidney injury after PCI remains debatable. It is likely that acute kidney injury after PCI is multifactorial and it may be preferable to use terms that do not assume that all renal dysfunction after PCI is secondary to contrast media. However, regardless of the term used, our study suggests that there is strong association between a high contrast dose and acute renal failure post PCI, even in high risk patients, implying that contrast media have an important contributory role towards the development of acute renal failure post PCI observed in this population. Only one post –procedure creatinine value was available and no follow up beyond the initial hospitalization was performed. Moreover, we lacked information on the timing of post-procedure serum creatinine measurements. No data on the type and amount of hydration used were available and this likely varied across institutions. However, we believe this makes our findings more generalizable to routine clinical care since it reflects observations from contemporary practice across multiple institutions.

To conclude, the use of a high contrast dose at time of PCI is associated with increased risks of CIN and NRD in all patients, regardless of their baseline predicted risk of these complications and renal function. Future research is needed to study whether the use of CV restricting measures decrease the risk of renal complications post PCI in patients with acute myocardial infarction. Until then, efforts to reduce CVs to less than thrice a patient’s CCC should be encouraged for all patients undergoing PCI.
